# SNP-based heritability estimates of the personality dimensions and polygenic prediction of both neuroticism and major depression: findings from CONVERGE

**DOI:** 10.1038/tp.2016.177

**Published:** 2016-10-25

**Authors:** A R Docherty, A Moscati, R Peterson, A C Edwards, D E Adkins, S A Bacanu, T B Bigdeli, B T Webb, J Flint, K S Kendler

**Affiliations:** 1 Departments of Psychiatry and Human Genetics, University of Utah School of Medicine, Salt Lake City, UT, USA; 2 Virginia Institute for Psychiatric and Behavioral Genetics, Virginia Commonwealth University School of Medicine, Richmond, VA, USA; 3 Department of Psychiatry and Biobehavioral Sciences, UCLA David Geffen School of Medicine, Los Angeles, CA, USA

## Abstract

Biometrical genetic studies suggest that the personality dimensions, including neuroticism, are moderately heritable (~0.4 to 0.6). Quantitative analyses that aggregate the effects of many common variants have recently further informed genetic research on European samples. However, there has been limited research to date on non-European populations. This study examined the personality dimensions in a large sample of Han Chinese descent (*N*=10 064) from the China, Oxford, and VCU Experimental Research on Genetic Epidemiology study, aimed at identifying genetic risk factors for recurrent major depression among a rigorously ascertained cohort. Heritability of neuroticism as measured by the Eysenck Personality Questionnaire (EPQ) was estimated to be low but statistically significant at 10% (s.e.=0.03, *P*=0.0001). In addition to EPQ, neuroticism based on a three-factor model, data for the Big Five (BF) personality dimensions (neuroticism, openness, conscientiousness, extraversion and agreeableness) measured by the Big Five Inventory were available for controls (*n*=5596). Heritability estimates of the BF were not statistically significant despite high power (>0.85) to detect heritabilities of 0.10. Polygenic risk scores constructed by best linear unbiased prediction weights applied to split-half samples failed to significantly predict any of the personality traits, but polygenic risk for neuroticism, calculated with LDpred and based on predictive variants previously identified from European populations (*N*=171 911), significantly predicted major depressive disorder case–control status (*P*=0.0004) after false discovery rate correction. The scores also significantly predicted EPQ neuroticism (*P*=6.3 × 10^−6^). Factor analytic results of the measures indicated that any differences in heritabilities across samples may be due to genetic variation or variation in haplotype structure between samples, rather than measurement non-invariance. Findings demonstrate that neuroticism can be significantly predicted across ancestry, and highlight the importance of studying polygenic contributions to personality in non-European populations.

## Introduction

Personality dimensions predict numerous social and behavioral outcomes (In the current sample, personality dimensions were predictive of education, marital status and employment status; refer to Supplementary Tables 1–3) and the Big Five (BF) were accepted as valid and meaningful higher-order factors by the 1990s.^1, 2, 3^ Of the dimensions, neuroticism in particular is a highly significant risk factor for psychiatric disorders and psychiatric comorbidity,^4, 5, 6, 7, 8^ as well as a host of physical health problems (for example, heart disease, eczema, asthma and irritable bowel syndrome^9, 10, 11^). Neuroticism also predicts response to treatment for both mental and physical health problems^12^ and generates significant economic burden.^13^ Hence, research developing a biological understanding of the personality dimensions has strong relevance for public health and for the prevention of mental health disorders.^14^

The BF personality dimension phenotypes appear to be highly polygenic, and may be most appropriate to explore with quantitative analyses that can aggregate the effects of many common single-nucleotide polymorphisms (SNPs; for example, genome-wide complex trait analysis or GCTA). Several twin and family (biometrical) studies estimated significant heritabilities for the BF personality dimensions on the order of ~0.4 to 0.6 (refs 15, 16, 17, 18) and negligible variance owing to shared environment^19^ (for a review, see Krueger and Johnson^20^). However, despite the apparent role of biological factors in the etiology of the personality dimensions, genome-wide association studies (GWAS) have only observed a small number of SNPs significantly associated with any of the BF.^21, 22, 23, 24^

In the largest genome-wide meta-analysis to date,^24^ neuroticism scores across >170 000 primarily European samples from the UK Biobank were harmonized and common variation from these GWAS explained up to 15% of the genetic variance in neuroticism.^23, 24^ These results suggest that adequately powered samples can detect modest yet noteworthy aggregate molecular genetic effects on neuroticism in Europeans.

However, to date, there has been no research aggregating common variation in large non-European samples. The current study examined heritabilities of the personality dimensions in the large China, Oxford and VCU Experimental Research on Genetic Epidemiology (CONVERGE) study of individuals of Han Chinese descent (*N*=10 064). This sample is substantial enough for analyses of common genetic variation (power=0.93 for heritability of 0.10), and evidences comparable rates of neuroticism. CONVERGE is the first study to identify and successfully replicate genome-wide significant loci hits for major depression,^25^ and this sample has other strengths as well: carefully ascertained Han Chinese ancestry allows for examination of whether genetic variation differs in samples of non-European descent compared with previously ascertained European samples, and the CONVERGE sample is female. Rates of major depressive disorder (MDD) in women are approximately twice that of men (cross-culturally and across diagnostic scheme or interview method^26, 27^) and an all-female sample is optimal because it controls for clinical heterogeneity owing to sex. Finally, CONVERGE used a clinically rich protocol with personality and interview-based diagnostic data on over 12 000 participants.

This study reflects a personality trait-based approach to the study of psychiatric disorders, consistent with the National Institute of Mental Health's Research Domain Criteria initiative to look beyond dichotomous, diagnostic status to identify core processes implicated in the development and maintenance of psychological disorders.^28^ Here, we estimate the heritabilities of neuroticism and the other personality dimensions in CONVERGE using GCTA, to see whether thousands of common genetic variants can explain variance in neuroticism or other BF traits. We then test the prediction of traits in split-half samples, by deriving best linear unbiased prediction (BLUP) scores,^29, 30^ with the intention of testing whether variants predicting neuroticism also predict MDD status, as has been found using European samples. Finally, we calculate polygenic risk scores for neuroticism based on the European sample (*N*>170 000) meta-analysis summary statistics, to predict both MDD status and neuroticism in CONVERGE. Scoring weights based on a subsample were recently found to significantly predict MDD status and neuroticism in independent cohorts of European ancestry.^23^

## Materials and methods

### Sample ascertainment

Cases and controls were recruited from 51 mental health centers and psychiatric departments of general medical hospitals, in 40 cities across 21 provinces. Please refer to previously published research for full details of sample ascertainment.^25^ We controlled for potential clinical heterogeneity by recruiting only female participants, and to control for ethnic stratification, only participants whose grandparents (all four) were of Han Chinese descent were recruited to participate. Cases and controls (age *M* (*s.d.*)=44.4 (8.9) and 47.7 (5.6), respectively) were excluded for diagnosis of bipolar disorder, any psychosis and any significant mental deficit such as a diagnosis of intellectual disability. Cases had to have had at least two major depressive episodes with the first episode occurring before age 50, and could not have abused drugs or alcohol before their first episode of depression. Controls were clinically screened to rule out prior depressive episodes and had to be at least 40 years of age, past the age of most typical MDD onset.

The study protocol was approved centrally by the Ethical Review Board of Oxford University, and by the ethics committees of all of the participating hospitals in China. All the participants provided written informed consent.

### DNA sequencing

DNA extraction, sequencing, genotyping and imputation details have been reported.^24^ Briefly, the DNA was extracted from saliva using Oragene and sequenced reads were obtained from Illumina Hiseq machines aligned to Genome Reference Consortium Human Build 37 patch release 5 (GRCh37.p5) with Stampy (v1.0.17; ref. 31) with default parameters. The reads consisting of base quality ⩽5 or containing adaptor sequencing were filtered out. The alignments were indexed in the BAM format^31^ using Samtools (v0.1.18; ref. 32) and PCR duplicates were marked for downstream filtering using Picardtools (v1.62). The Genome Analysis Toolkit's (GATK, version 2.6; ref. 33) BaseRecalibrator created recalibration tables to screen known SNPs and INDELs in the BAM files from dbSNP (version 137, excluding all sites added after version 129). GATKlite (v2.2.15) was used for subsequent base quality recalibration and removal of read pairs with improperly aligned segments as determined by Stampy.

### Calling and imputation of genotypes

GATK's UnifiedGenotyper (version 2.7-2-g6bda569) VariantRecalibrator (version 2.7-2-g6bda569) were used on post subsequent base quality recalibration files for variant discovery and genotyping at all polymorphic SNPs in 1000 G Phase 1 ASN panel^34^ as well as variant quality score recalibration. A sensitivity threshold of 90% was applied for imputation after optimizing for Transition to Transversion ratios. Genotype likelihoods were calculated using a binomial mixture model implemented in SNPtools (version 1.0)^35^ and imputation was performed at sites with no reference panel using BEAGLE (version 3.3.2).^36^ A second round of imputation was performed at biallelic polymorphic SNPs using 1000 G Phase 1 ASN haplotypes as a reference panel. To determine the final number of SNPs, we applied a conservative inclusion threshold for SNPs: (1) a *P*-value for violation *HWE*>10-6, (2) information score >0.9 and (3) minor allele frequency in CONVERGE >0.5%.

### Diagnostic and personality assessments

All the participants were interviewed using a computerized assessment system and the participant interview sessions lasted approximately 2 h. The interviewers were largely trained psychiatrists with a small number representing postgraduate medical students or psychiatric nurses, and all were clinically trained by the CONVERGE team for at least 1 week. The interviews were recorded and included an assessment of psychopathology, demographic characteristics and psychosocial functioning. Trained editors listened to a portion of the interviews to provide ratings of interview quality. We excluded participants who had incomplete assessment information or were lacking high-quality genetic data, to arrive at a final 4728 controls and 5612 case samples for analysis.

During the interview, control the participants completed the Big Five Inventory (BFI)^37, 38^ which had been developed in English and translated into Mandarin. The measure consists of 44 items rated on a 1–5 Likert Scale (strongly disagree to strongly agree) and subscales are scored for each of the five BF traits.

The control and case samples completed the Neuroticism subscale of the Eysenck Personality Questionnaire,^39^ a 40-item true–false self-report scale. This assessment was also developed in English and translated to Mandarin. Because the Mandarin version of the BFI had not previously been examined for internal reliability, all translated scales were examined carefully and any items with poor subscale factor loadings were removed from the subscale calculations. For example, one of the BFI Openness items generated a negative factor loading, thus analyses of this subscale were run with and without the poorly performing item. Before removing any items, both BFI and Eysenck Personality Questionnaire (EPQ) assessments generally showed adequate to high reliability, and Cronbach's alphas for each of the the scales in this sample are presented in Table 1. One BFI Openness and three BFI Extraversion items with poor loadings were removed, resulting in an improvement in reliability.

### Population structure

To reduce the effects of population stratification, ancestry principal components were constructed using EIGENSOFT 3.0 (ref. 40) and SMARTPCA.^41^ To correct for dependence between markers, and thereby avoid the potential disruption of the eigenvalue structure, SNPs were pruned at *r*^2^>0.7 before construction of principal component scores, as recommended by Patterson *et al.*^41^ The principal component analysis was conducted to obtain principle components of population stratification. A total of 144 929 autosomal SNPs with Pr(G)⩾0.9 and <1% missing rate were used to generate 10 intracontinental principal component scores. To circumvent over-fitting,^40, 41, 42^ only the first two principal components, which distinguished north–south regional differences, were used in the subsequent analyses.

### SNP-based heritability

The GWAS methods can be complimented by GREML (genomic relatedness restricted maximum likelihood) methods, namely genome-wide complex trait analysis (GCTA). Genomic relatedness analyses integrate and test the effect of variation across all genotyped variants. The use of genomic relatedness data have been informative across many disorders where univariate genome-wide association tests have been unsuccessful in accounting for a significant proportion of genetic variance (for example, autism, Parkinson's disease, affective disorders^43, 44, 45^). GCTA-based methods can examine information from all genotyped DNA in one analysis, providing a genome-based approach to quantify the heritability of complex psychiatric phenotypes.^29, 46^

For each analysis presented, the GCTA package^29, 46, 47^ was used to create a genetic relatedness matrix file containing identity by state relationship calculations for all pair-wise sets of individuals. The REML analysis was then performed in GCTA using the respective genetic relatedness matrices and the quantitative principal component covariates. An estimate of relatedness is used as a random effect in a mixed linear model when predicting phenotypic relatedness by restricted maximum likelihood, resulting in an estimate of the variance in the trait owing to all genotyped SNPs. GCTA analyses of the BFI traits were performed within the control sample, and similar analyses of EPQ neuroticism were performed across the entire sample of controls and cases. EPQ neuroticism was then examined in the controls and cases separately. Covariates included the two primary principle components. Power calculations using the GREML-GCTA power calculator created by Hermani and Yang (for details, see Visscher *et al.*^43^) indicated greater than 85% power to detect heritability estimates of 0.20.

### Genetic risk score prediction of the personality dimensions based on split-half samples

Genetic risk scores were constructed using estimated SNP effects by the BLUP method^29^ using the first random half of the sample and then testing the aggregate score in the remaining half. We then reversed the analysis to predict scores in the first half of the sample from the second half. Linear regressions were conducted for each dimension including a full model (BLUP score and the two primary principle components as predictors of the trait) and a restricted model, removing the BLUP score. The difference in Nagelkerke *R*^2^ between models (rsq) was computed for each trait and the *P*-value associated with the SNP score variable within the full model was examined. The Nagelkerke rsq generated from these models is a difference in pseudo-*R*^2^, thus *P*-values are not derived from full and restricted model comparisons. Instead, the *P*-value associated with the dropped component (score) is reported here.

### Polygenic risk for neuroticism based on European samples as a predictor of MDD and neuroticism in converge

Analyses also generated polygenic risk profile scores based on weights from previous meta-analyses that had successfully predicted MDD and neuroticism in independent cohorts.^23, 24^ Polygenic risk profile scores calculated from the UK Biobank summary statistics were used to test predictive power in CONVERGE. LDpred^48^ was the preferred method for these analyses, owing to the ability of LDpred to account for linkage disequilibrium (LD) structure using our EUR test sample, and because *P*-value thresholds do not need to be specified using LDpred, nor do variant lists need to be pruned for LD. The method does require prior proportions of causal variants in the genome to be assumed for score calculations, and a range (proportions of 1, 0.3, 0.1, 0.03, 0.01, 0.003 and 0.001, as well as the model of infinite variants of infinitesimally small effect^49^) was tested to avoid making errors based on incorrect theory. Regressions were run using R to compare full (RPS, ancestry principle components) and restricted models where RPS was removed.

RPSs were used to predict both MDD status (binary case–control) and neuroticism (quantitative EPQ score) in CONVERGE. For these analyses, neuroticism was examined as both a sum score and a factor score, but results did not appreciably differ across the two types of scores.

## Results

Characteristics of the trait distributions for the BFI are listed in Table 2, and distributions indicated normality. The neuroticism subscale of the EPQ exhibited skew and kurtosis within the control sample and the scores from the entire sample were square root transformed, though analytic results did not appreciably differ with or without this transform.

Heritability estimates (*σ*^2^_G_*/σ*^2^_P_) of the BFI scales in the controls are presented in Table 3. Likelihood ratio tests were one-sided. Agreeableness exhibited a heritability estimate trending toward significance (*P*=0.06) and none of the other subscales exhibited statistically significant heritability estimates. This was true even when adjusting starting values (the —reml-priors function in GCTA) and increasing the number of iterations (the —reml-maxit function in GCTA).

Table 4 presents the same information for the EPQ neuroticism subscale in the entire sample (the correlation between the two neuroticism subscales was 0.5, *P*<0.0001). This estimate, at 10% with 3% standard error, was statistically significant. For this sample, power was approximately 0.93 to detect a heritability of 0.10. The controls and cases were also examined separately, with heritability analyses resulting in nonsignificant estimates.

Polygenic prediction of the BF using BLUP scores was then conducted. Table 5 presents analyses predicting the dimensions from BLUP scores in split-half samples. Individual variants of a first sample (Group 1) were used to predict the trait in a second sample (Group 2). Tests were conducted using Group 1 as a training sample and Group 2 as a test sample, and then predictive analyses were reversed. None of the BLUP scores successfully predicted EPQ neuroticism in a second sample (examining cases, controls or the entire sample) and none of the BF dimensions were significantly predicted from BF BLUP scores in the control sample.

Finally, we examined RPSs based on the sample weights from discovery samples that had significantly predicted MDD and neuroticism in independent cohorts of European ancestry. Polygenic risk of neuroticism, based on the discovery set^24^ and calculated by LDpred, was significantly predictive of MDD status and neuroticism in CONVERGE at a prior threshold of 0.3 after false discovery rate correction (for MDD, rsq=0.001, *P*=0.00047; for neuroticism, rsq=0.083, *P*=6.34 × 10^−6^). Risk profile scores did not significantly predict BFI neuroticism (only available in the Han controls).

## Discussion

The current study examined common genomic variation in relation to the major dimensions of personality. Results suggest that when examining a sufficiently large sample, EPQ neuroticism can be successfully predicted from common variants with a genome-based heritability of approximately 10% in individuals of Han Chinese ancestry. When examining controls separately using the BFI, common genetic variants appeared to have a negligible effect on personality dimensions.

Although scientific consensus converged on three- and five-factor dimensional models of personality decades ago, research has only now begun to successfully link these dimensions to molecular genetic variants. Both GPC and UK Biobank analyses suggest shared genetic architecture of MDD and EPQ neuroticism. This is supported by successful polygenic risk prediction of neuroticism and MDD status from discovery sample statistics (including samples across Europe, USA and Australia^23, 24^), and is consistent with genetic correlations found in twin research.^50, 51^

Individual risk loci for neuroticism were not replicated in GPC analyses,^23^ but new neuroticism susceptibility loci were identified in a meta-analysis of UK Biobank, the Generation Scotland: Scottish Family Health Study, and Queensland Institute of Medical Research cohorts, using the same neuroticism measure analyzed here.^52^ In addition, Benjamin and colleagues reported significant pathway enrichment in the sample when considering neuroticism, MDD and subjective well-being jointly.^53^ Future research could attempt to replicate these findings in a non-European sample by examining candidate loci and pathway enrichment in CONVERGE.

Other attempts to identify risk loci have found SNPs to be associated with openness and conscientiousness dimensions, but these results failed later replication attempts. Overall, single SNP effects derived from univariate GWAS methods have accounted for a very small proportion of the variance in these dimensions in samples of largely European ancestry.^22, 52, 54^

Attention has been increasingly drawn to the examination of more complex genetic architectures in the study of human personality and behavioral traits.^55, 56, 57, 58^ Results from GREML studies are consistent with the small effects produced by SNPs found in GWAS of personality, and these studies have, to date, always produced somewhat low heritability estimates.^54, 56, 58^ The results from our well-powered study serve to further support generally low heritability estimates of the personality dimensions based on common genetic variants.

It remains unclear why heritability estimates from genome-based studies, including this one, are largely inconsistent with the increased heritability estimates derived from twin studies. One explanation for decreased genome-based estimates could be that these dimensions are associated with rare variants, insertions, deletions or other types of genomic variation. It is also possible that twin studies overestimate narrow sense heritability and underestimate the variance due to nonadditive genetic and common environmental factors. Twin research by Loehlin *et al.*^59^ has suggested that nonadditive genetic effects may be especially relevant to personality traits (for a discussion of nonadditive genetic effects in personality research; see Lykken *et al.*^60^). This is not inconsistent with the previous univariate GWAS findings or with lower estimates reported from studies using adoption designs.^61, 62, 63^ Non-additive genetic variance could also be less relevant for neuroticism compared with, for example, extraversion, accounting for the lower SNP-based *h*^2^ estimates for extraversion and the other BF traits. This could contribute to the differences between the 10–15% and the ~40–50% heritability observed in biometrical studies of neuroticism.

Although all of these explanations are possible and by no means mutually exclusive, we believe it is likely the case that the effect sizes of the individual variants on personality are likely to be very small. Although our sample was relatively large, much larger samples have been necessary to detect the very small additive genetic effects of individual molecular variants on personality.

It is somewhat incongruous that significant *h*^2^ was detected for neuroticism in the entire sample, yet polygene signals in split-halves were not significantly predictive of neuroticism. This may be a statistical artifact of the case–control design used in CONVERGE, and the differential distributions of neuroticism that were observed across the MDD and control samples. Another possibility is that reduced power from splitting the sample, skewness or zero-inflation in the neuroticism distribution led to these results.

In addition, some generalizability concerns are relevant to this study. First, though examination of only females controlled for sex-related clinical heterogeneity, it is possible that this led to different point estimates than would be found with males and females. This would be consistent with previous research examining sex differences in genome-based estimation of heritability of neuroticism and extraversion in males and females.^56^ The rate of MDD in women is approximately twice that of men^26, 27^ and longitudinal studies indicate that this sex difference first emerges mid-puberty,^64, 65, 66, 67, 68, 69^ which could implicate dynamic epigenetic, endocrine and/or environmental changes.

Second, the trait scales used were originally developed in English, and although reliability of the scales in this sample was generally adequate, not all of the translated items performed well. However, although measurement non-invariance across European and Chinese samples should be further explored, factor analytic results of the measures suggest that differences in heritabilities across samples may be owing to genetic variation or variation in haplotype structure between samples.

Finally, general measurement issues relating to the BF may benefit from further scrutiny. Two items on the BFI corresponding to remaining calm in stressful situations (a potential facet of neuroticism that is not directly measured by the EPQ) were the least correlated with EPQ items. This difference between the scales could account for discrepant *h*^2^ results across measures. It is also important to point out that the five factors have been critiqued as only partially empirically derived.^70, 71, 72, 73^ Some have speculated that the five personality dimensions may be statistical artifacts of the factor analytic methods used. Common pathway modeling has indicated overlap of these constructs, with a general violation of collinearity constraints (for a helpful review, see Franic *et al.*^74^).

Results from this well-powered study suggest that with a sufficiently large sample, neuroticism based on the EPQ can be successfully predicted from common genetic variants. Overall, common genetic variation appeared to have a very limited effect on the major dimensions of personality in our sample of controls. Conceivably, a clinically enriched sample with MDD (and thus with greater mean levels of neuroticism) could enrich the burden of relevant alleles in the sample, increasing power to detect risk variants. Nevertheless, the use of both a healthy control group and a depressive disorder case group in this study separately produced low and nonsignificant heritability estimates. Future genome-based personality studies would likely benefit from the use of larger samples, and given the paucity of significant, replicated GWAS association findings, future research on the genetics of personality may also benefit from further use of gene-based and gene pathway methods to examine enrichment relating to personality dimensions.

## Figures and Tables

**Table 1:** Personality trait descriptive statistics and Cronbach's alphas

	*Mean (s.d.)*	*Median*	*Min*	*Max*	*Range/possible*	α	*Lower, upper CIs*
*BFI (*n=*5596)*						0.86	0.85, 0.86
Neuroticism	19.81 (4.98)	20	8	39	31/32	0.70	0.68, 0.71
Extraversion	19.07 (3.42)	19	9	40	31/32	0.70	0.69, 0.70
Openness	28.86 (6.31)	29	10	49	39/40	0.80	0.80, 0.81
Conscientiousness	34.13 (4.72)	34	14	45	31/36	0.67	0.66, 0.68
Agreeableness	36.64 (4.45)	37	17	45	29/36	0.67	0.65, 0.68
							
*EPQ neuroticism*	8.15 (6.87)	7	0	23	23/23	0.93	0.93, 0.94
Controls (*n*=5596)	3.39 (3.87)	2	0	23	23/23	0.85	0.85, 0.86
Cases (*n*=5612)	12.72 (5.96)	13	0	22	22/23	0.89	0.88, 0.89

Abbreviations: *α*, Cronbach alpha; BFI, Big Five Inventory; CIs, 95% confidence intervals; EPQ, Eysenck Personality Questionnaire.

Range/possible is the range observed in the data relative to the maximum possible range of scores. One negatively loading item was removed from the BFI openness subscale, and three poorly loading items (<0.30) were removed from the BFI extraversion subscale.

**Table 2:** Distributional characteristics of the personality dimension scores

	*Skew*	*Kurtosis*
*BFI*		
Neuroticism	−0.02	−0.01
Extraversion	0.03	0.08
Openness	−0.27	0.14
Conscientiousness	0.04	−0.25
Agreeableness	−0.26	−0.10
EPQ neuroticism	−0.48	−1.06
*EPQ neuroticism transform*	−0.26	−1.06
Cases	−0.27	−0.89
Cases transform	−1.04	0.92
Controls	1.59	2.55
Controls transform	0.26	−0.70

Abbreviations: BFI, Big Five Inventory; EPQ, Eysenck Personality Questionnaire; Transform, EPQ neuroticism square root transform.

**Table 3:** SNP-based BFI heritabilities in controls (*n*=4728)

	σ^*2*^_*G*_ *(s.e.)*	σ^*2*^_*G*_*/σ*^*2*^_*P*_ *(s.e.)*	*LRT (df=1)*	P
Neuroticism	<0.001 (1.45)	<0.001 (0.05)	<0.001	0.5
Extraversion	<0.001 (0.68)	<0.001 (0.06)	<0.001	0.5
Openness	1.14 (2.32)	0.03 (0.06)	0.25	0.3
Conscientiousness	1.20 (1.32)	0.05 (0.06)	0.85	0.2
Agreeableness	1.87 (1.18)	0.10 (0.06)	2.50	0.06

Abbreviations: BFI, Big Five Inventory; df, degrees of freedom; LRT, likelihood ratio test; *P*, *P*-value for the LRT; SNP, single-nucleotide polymorphism; *σ*^2^_G_, genetic variance; *σ*^2^_G_*/σ*^2^_P_, proportion of total variation due to common variants.

**Table 4:** SNP-based EPQ neuroticism heritabilities in entire sample of cases and controls

	σ^*2*^_*G*_ *(s.e.)*	σ^*2*^_*G*_*/*σ^*2*^_*P*_ *(s.e.)*	*LRT (df=1)*	P
Entire sample (*n*=9633)	4.79 (1.39)	0.10 (0.03)	13.65	0.0001
Controls (*n*=4820)	0.99 (0.88)	0.07 (0.06)	1.28	0.13
Cases (*n*=4813)	2.88 (2.05)	0.08 (0.06)	2.03	0.08

Abbreviations: EPQ, Eysenck Personality Questionnaire; df,degrees of freedom; LRT, likelihood ratio test; *P*, *P*-value for the LRT; SNP, single-nucleotide polymorphism; *σ*^2^_G_, genetic variance; *σ*^2^_G_*/σ*^2^_P_, proportion of total variation due to common genetic variants.

**Table 5:** Prediction of EPQ neuroticism and BFI personality dimensions in split-half samples using BLUP scores

	*BLUP rsq*	P
*EPQ neuroticism*		
*Combined (*n=*9633)*		
Group 1	<0.001	>0.99
Group 2	<0.001	0.09
*Cases (*n=*4813)*		
Group 1	0.002	0.18
Group 2	0.002	0.26
*Controls (*n=*4820)*		
Group 1	0.001	0.53
Group 2	<0.001	0.66

*BFI (Controls;* n=*4728)*		
*Neuroticism*		
Group 1	<0.001	0.93
Group 2	<0.001	0.98
*Extraversion*		
Group 1	0.01	0.24
Group 2	0.004	0.46
*Openness*		
Group 1	<0.001	0.87
Group 2	<0.001	0.85
	*Conscientiousness*		
Group 1	0.01	0.23	
Group 2	0.02	0.20	
	*Agreeableness*		
Group 1	0.007	0.35	
Group 2	0.009	0.29	

Abbreviations: BFI, Big Five Inventory; BLUP, best linear unbiased predictor; EPQ, Eysenck Personality Questionnaire; *P*, *P*-value of the BLUP score component of the full model; rsq, difference in Nagelkerke pseudo-*R*^2^ between the full model including BLUP score and the two primary components, and the restricted model dropping the BLUP score.
