# Supplementary material for: SNP-based heritability estimates of the personality dimensions and polygenic prediction of both neuroticism and major depression: findings from CONVERGE
Source: Transl Psychiatry. 2016 Oct 25;6(10):e926–. doi: 10.1038/tp.2016.177 (PMC5290344; doi:10.1038/tp.2016.177)
Supplement: Supplementary Table 1 [file tp2016177x1.docx]

| Supplementary Table 1. *Big Five Inventory Subscales by Marital Status in Controls (N = 5588)* | | | | | | | |  |
| --- | --- | --- | --- | --- | --- | --- | --- | --- |
|  | Married | Separated | Divorced | Widowed | Never Married | *F_(4,5587)_* |  |  |
| Sample *n* | 5170 | 34 | 182 | 168 | 35 |  |  |  |
| Neuroticism | 19.81 | 20.85 | 19.66 | 19.61 | 19.20 | 0.57 |  |  |
| Extraversion | 27.24 | 26.41 | 27.37 | 27.02 | 26.20 | 0.86 |  |  |
| Openness | 31.05 | 33.29 | 33.59 | 31.24 | 34.69 | 21.56*** |  |  |
| Conscientiousness | 34.11 | 33.76 | 34.66 | 34.21 | 35.03 | 2.08 |  |  |
| Agreeableness | 36.65 | 36.85 | 36.55 | 36.59 | 36.34 | 0.17 |  |  |
| *** *p* ≤.0001 | | | | | | | |  |
