# Supplementary material for: SNP-based heritability estimates of the personality dimensions and polygenic prediction of both neuroticism and major depression: findings from CONVERGE
Source: Transl Psychiatry. 2016 Oct 25;6(10):e926–. doi: 10.1038/tp.2016.177 (PMC5290344; doi:10.1038/tp.2016.177)
Supplement: Supplementary Table 2 [file tp2016177x2.docx]

| Supplementary Table 2. *Big Five Inventory Subscales by Level of Education in Controls (across 11 categories; N = 3909)* | |  |
| --- | --- | --- |
|  | *F_(10,3908)_* |  |
| Neuroticism | 35.83*** | |
| Extraversion | 2.34 | |
| Openness | 382.90*** | |
| Conscientiousness | 79.81*** | |
| Agreeableness | 24.11*** | |
| *** *p* ≤.0001.  *Note*: Education was positively associated with Openness, Conscientiousness, Extraversion, and Agreeableness, and negatively associated with Neuroticism. | |  |
|  |  |  |
