# Supplementary material for: SNP-based heritability estimates of the personality dimensions and polygenic prediction of both neuroticism and major depression: findings from CONVERGE
Source: Transl Psychiatry. 2016 Oct 25;6(10):e926–. doi: 10.1038/tp.2016.177 (PMC5290344; doi:10.1038/tp.2016.177)
Supplement: Supplementary Table 3 [file tp2016177x3.docx]

| Supplementary Table 3. *Big Five Inventory Subscales by Employment in Controls (N = 3911)* | | | | | | | |
| --- | --- | --- | --- | --- | --- | --- | --- |
|  | Work/  School | Home-maker | Retired | Other | Laid off/  Disabled | Job-Seeking | *F_(5,3910)_* |
| Sample *n* | 1972 | 755 | 707 | 311 | 137 | 30 |  |
| Neuroticism | 19.78 | 20.56 | 19.22 | 20.64 | 20.18 | 20.37 | 11.56*** |
| Extraversion | 27.07 | 27.14 | 27.99 | 26.99 | 26.28 | 25.93 | 1.04 |
| Openness | 32.02 | 29.34 | 32.39 | 27.69 | 30.87 | 32.63 | 163.30*** |
| Conscientiousness | 34.32 | 33.32 | 34.45 | 33.74 | 33.84 | 32.80 | 15.99*** |
| Agreeableness | 36.66 | 36.35 | 36.92 | 35.75 | 36.19 | 35.93 | 6.82** |
| ***p* <.01  *** *p* ≤.0001 | | | | | | | |
